# Evaluating cochlear insertion trauma and hearing preservation after cochlear implantation (CIPRES): a study protocol for a randomized single-blind controlled trial

**DOI:** 10.1186/s13063-021-05878-2

**Published:** 2021-12-09

**Authors:** Saad Jwair, Ralf A. Boerboom, Huib Versnel, Robert J. Stokroos, Hans G. X. M. Thomeer

**Affiliations:** 1 Department of Otorhinolaryngology and Head & Neck Surgery, University Medical Center Utrecht, Utrecht University, P.O. Box 85500, 3508 GA Utrecht, the Netherlands; 2 UMC Utrecht Brain Center, Utrecht University, Utrecht, the Netherlands

**Keywords:** Cochlear implant, Insertion trauma, Electrode array, Lateral wall, Perimodiolar, Round window, Cochleostomy, Scalar translocation, Electrocochleography

## Abstract

**Background:**

In order to preserve residual hearing in patients with sensorineural hearing loss (SNHL) who receive a cochlear implant (CI), insertion trauma to the delicate structures of the cochlea needs to be minimized. The surgical approach comprises the conventional mastoidectomy-posterior tympanotomy (MPT) to arrive at the middle ear, followed by either a cochleostomy (CO) or the round window (RW) approach. Both techniques have their benefits and disadvantages. Another important aspect in structure preservation is the design of the electrode array. Two different designs are used: a “straight” lateral wall lying electrode array (LW) or a “pre-curved” perimodiolar lying electrode array (PM). Interestingly, until now, the best surgical approach and design of the implant is uncertain. Our hypothesis is that there is a difference in hearing preservation outcomes between the four possible treatment options.

**Methods:**

We designed a monocenter, multi-arm, randomized controlled trial to compare insertion trauma between four groups of patients, with each group having a unique combination of an electrode array type (LW or PM) and surgical approach (RW or CO). In total, 48 patients will be randomized into one of these four intervention groups. Our primary objective is the comparison of postoperative hearing preservation between these four groups. Secondly, we aim to assess structure preservation (i.e., scalar translocation, with basilar membrane disruption or tip fold-over of array) for each group. Thirdly, we will compare objective outcomes of hearing and structure preservation by way of electrocochleography (ECochG).

**Discussion:**

Cochlear implantation by way of a cochleostomy or round window approach, using different electrode array types, is the standard medical care for patients with severe to profound bilateral sensorineural hearing loss, as it is a relatively simple and low-risk procedure that greatly benefits patients. However, loss of residual hearing remains a problem. This trial is the first randomized controlled trial that evaluates the effect of cochlear insertion trauma of several CI treatment options on hearing preservation.

**Trial registration:**

Netherlands Trial Register (NTR) NL8586. Registered on 4 May 2020. Retrospectively registered; 3/48 participants were included before registration.

**Supplementary Information:**

The online version contains supplementary material available at 10.1186/s13063-021-05878-2.

## Administrative information

Note: the numbers in curly brackets in this protocol refer to SPIRIT checklist item numbers. The order of the items has been modified to group similar items (see http://www.equator-network.org/reporting-guidelines/spirit-2013-statement-defining-standard-protocol-items-for-clinical-trials/).

**Table:** 

Title {1}	Evaluating cochlear insertion trauma and hearing preservation after cochlear implantation (CIPRES): A study protocol for a randomised single blind controlled trial
Trial registration {2a and 2b}.	Netherlands Trial Register (www.trialregister.nl): NL8586
Protocol version {3}	Protocol Version 3, 02-01-2020
Funding {4}	Advanced Bionics funds this study in total by providing the funds for the PhD project of the main author (SJ) and funding of necessary laboratory material.
Author details {5a}	List of authors: -Saad Jwair^12^, s.jwair@umcutrecht.nl, corresponding author -Ralf A. Boerboom^12^ -Huib Versnel^12^ -Robert J. Stokroos^12^ -Hans G.X.M Thomeer^12^ ^1^ Department of Otorhinolaryngology and Head & Neck Surgery, University Medical Center Utrecht, Utrecht University, P.O. Box 85500, 3508 GA, Utrecht, the Netherlands ^2^ UMC Utrecht Brain Center, Utrecht University, the Netherlands
Name and contact information for the trial sponsor {5b}	UMC Utrecht, Heidelberglaan 100, 3584 CX Utrecht, The Netherlands, telephone number: 0031-887569476
Role of sponsor {5c}	The sponsor (UMC Utrecht) designs and executes the trial. The funding sources had no role in the design and conduct of the study; collection, management, analysis, and interpretation of the data; preparation, review, or approval of the manuscript; and decision to submit the manuscript for publication.

## Introduction

### Background and rationale {6a}

In people with severe sensorineural hearing loss or deafness, hearing can be (partially) restored with a cochlear implant (CI). A CI bypasses the sensory hair cells and directly stimulates the auditory nerve via electrical current pulses, allowing deaf patients to hear again. Cochlear implantation has become a standard and accepted treatment for severely hearing impaired patients throughout the years in high-income countries. Hearing with a CI has seen a tremendous development in auditory perception, from only sound detection in the 1980s to speech understanding in the last decades [1]. However, speech understanding is far from optimal, especially in difficult situations where background noise is present, and perception of other sounds as music can also be quite troublesome. Several studies have shown that preserving residual hearing can lead to better hearing outcomes, especially in noisy environments [2–5]. In order to preserve residual hearing, trauma to the delicate structures of the cochlea needs to be minimized during the surgical implantation procedure.

The surgical procedure commonly starts with the conventional mastoidectomy-posterior tympanotomy (MPT) approach to the middle ear and is followed by accessing the cochlea, through either a cochleostomy (CO) or the round window (RW). Several papers, including systematic reviews comparing CO and RW approaches in the literature, concluded that evidence lacks regarding preference for one or the other approach with respect to hearing preservation [6–10]. Both techniques of accessing the cochlea have their potential pros and cons (e.g., cochleostomy leads to a smaller angle of insertion and by definition induces damage to the outer bony wall and spiral ligament, while the RW approach ensures a correct positioning of the electrode array and leaving the outer bony wall and spiral ligament intact). The extended round window (ERW) approach will not be tested in this study. The ERW approach is a combination of the direct RW and cochleostomy approach and is generally considered to be a variant of the cochleostomy approach. It is therefore unlikely that the ERW approach would be significantly different than the cochleostomy approach. One may also argue a preference for a certain approach based on individual cochlear structures. Several studies for example have clearly shown that each human cochlea has a different “cochlear hook” in parallel with one’s unique fingerprint [11–13].

Correct insertion, for both cochleostomy and round window approach, ensures that the implant is in the scala tympani of the cochlea [14]. If during insertion, the CI translocates to the scala vestibuli or scala media, the basilar membrane with the organ of Corti (the physiological receptor organ that transduces the acoustic energy) is damaged. Scalar translocation can negatively influence the final hearing outcome and hearing preservation of CI patients [15, 16].

Another aspect relevant for minimizing insertion trauma is the design of the electrode array. There are two fundamentally different designs: a “straight” lateral wall lying electrode array (LW) or a “pre-curved” perimodiolar lying electrode array (PM). No evidence has been provided that one design outperforms the other in terms of hearing with a CI and structure preservation [7, 10, 15]. On the one hand, lateral wall positioning might be the best way to preserve structures as the osseous spiral lamina and basilar membrane; on the other hand, perimodiolar positioning might provide better hearing with a CI (which is the ultimate objective for deaf patients with a CI), as the electrodes are situated closer to the medially situated spiral ganglion cells which form the auditory nerve and need to be electrically stimulated. In addition, the perimodiolar array has the potential to minimize contact between the array and the lateral wall, leading to structure preservation of the lateral wall and stria vascularis.

According to one study, speech perception scores were better for the LW group [17]. On the contrary, other studies report better speech perception outcomes for the PM group [7, 15]. The majority of the studies, however, showed no difference between both groups [18–22]. However, all these studies had a high risk of bias. In addition, the studies failed to differentiate between the surgical approaches, inducing a major confounding factor. Additionally, an interaction effect may be present with the effect of the electrode array type on the surgical approach and other outcomes being different for the two surgical approaches.

It is unclear which surgical approach and electrode design are most suited to achieve minimal insertion trauma, and thereby preserving residual hearing in cochlear implantation surgery. Therefore, it is not surprising that worldwide both type of approaches and electrode designs are used.

Considering the surgical approach and electrode array design, it is important to note that during insertion no reliable feedback is provided regarding the array tip position in relation to the intracochlear structures. After inserting the tip of the electrode array in the round window perforation or cochleostomy, only tactile feedback is available which might not be sufficient to distinguish whether the implant is correctly inserted.

One of the possibilities to view the intracochlear structures and thereby discern the scalar location of the array in relation to the micro-anatomical structures (thus providing postoperative feedback) is by applying imaging techniques after surgery such as cone beam computed tomography (CB-CT), which has been proven to be reliable in differentiating the different scalae and exact electrode array position [23–25]. Another possibility to detect insertion trauma is by intraoperative electrophysiological measurements, providing indirect feedback: intracochlear electrocochleography (ECochG) which measures responses of residual functioning hair cells and spiral ganglion cells to acoustic tone stimuli. During insertion, ECochG measures can be used to assess the probability of insertion trauma, thus providing feedback of the insertion [26–29].

### Objectives {7}

The primary objective of this study is to compare hearing preservation after cochlear implantation between the four possible combinations of surgical approaches (CO and RW) and electrode array designs (LW and PM). Hearing preservation will be measured postoperatively with pure tone audiometry. Secondary objectives are to compare the effect of these interventions on scalar position and ECochG measures. Furthermore, we aim to assess the relationship between the outcome measures for hearing preservation (audiometry, ECochG, and postoperative CT).

### Trial design {8}

This study concerns a single-blind, monocenter, multi-arm randomized trial. All four treatment options are implemented interchangeably in standard medical care. Our hypothesis is that there are differences in hearing preservation between these four treatment options. Participants will be blinded for surgical approach/type of electrode. In total, 48 participants will be included, all groups carry the same equal weight (allocation ratio 1:1:1:1). In case of a drop-out, a replacement will be included to ensure 48 participants who completed the study.

## Methods: participants, interventions, and outcomes

### Study setting {9}

This is a monocenter study performed at the Department of Otorhinolaryngology in the University Medical Center Utrecht, an academic hospital, and is expected to run for approximately 3 years.

### Eligibility criteria {10}

All participants will undergo the usual standard medical care of work-up before, during, and after cochlear implantation. The work-up includes a pure tone audiogram (PTA), a speech audiogram, a preoperative CT, and interviews with the speech therapist, audiologist, ENT surgeon, and social worker. In a multidisciplinary meeting, the Cochlear implantation team of the UMC Utrecht will assess all results and decide whether a patient is eligible for a CI. A patient is eligible if phoneme score (based on CVC words) with hearing aids ≤60% and/or speech perception with noise is insufficient according to criteria adopted by Snel-Bongers et al [30]. In addition, the personal expectations, beliefs, and motivation of the patient play an important role. In addition, according to standard medical care, participants will receive corticosteroids before and after surgery.

*Inclusion criteria*

-Dutch language proficiency

-18 years or older

-Choice for Advanced Bionics implant

-No signs of acute or chronic middle ear infections and/or mastoiditis

*Exclusion criteria*

-Prior otologic surgery in the implanted ear (excluding tympanostomy tube placement)

-Inner ear malformation (i.e., ossification, Mondini malformation)

-Retrocochlear pathology

-Neurocognitive disorders

-Acute or chronic otomastoiditis

### Who will take informed consent? {26a}

Participants ENT-physician or audiologist during visits to the outpatient clinic will ask whether the patient would be interested to participate in the study. Additional verbal and written information about the study will be provided to all participants by an investigator. An investigator will also provide and obtain the informed consent (IC) form, which is also co-signed by the investigator. There will be ample opportunity (at least 1 week) for the participants to consider participation and discuss their questions with one of the investigators before the participants may decide to sign the IC form in order to participate. Participation in the study is entirely voluntary. If a subject wants to participate, several appointments for the audiological follow-up will be scheduled. If a patient does not want to participate, contact with the investigator will be terminated.

### Additional consent provisions for collection and use of participant data and biological specimens {26b}

Not applicable, no additional consent is required for use of participant data and biological specimens.

## Interventions

### Explanation for the choice of comparators {6b}

Four groups of participants will be included, which all have a different combination of electrode type and surgical insertion approach. These treatment options are all standard care in cochlear implants centers worldwide.

### Intervention description {11a}

The electrode type consists of either a lateral wall electrode array or a perimodiolar electrode array, specifically, respectively, the SlimJ and Midscala electrode arrays. Both these arrays are developed by Advanced Bionics. Two surgical approaches are used, a round window or cochleostomy approach. The cochleostomy is placed antero-inferiorly from the round window niche.

### Criteria for discontinuing or modifying allocated interventions {11b}

It is only possible to change the allocated intervention via a second surgery, by removing the cochlear implant. A second surgery increases potential harm for the patient, outweighing potential benefits. Therefore, removal of the cochlear implant is only performed if medically necessitated, e.g., in instances of malfunctioning device, wound infection, or persisting pain. Such rare cases will be discussed in a plenary session dedicated for cochlear implant patients, in line with normal standard medical care.

### Withdrawal of individual subjects

Subjects can leave the study at any time for any reason if they wish to do so without any consequences. The investigator can decide to withdraw a subject from the study for urgent medical reasons.

### Strategies to improve adherence to interventions {11c}

To improve adherence to the study protocol, the follow-up measurements for the study are planned simultaneously with the standard medical rehabilitation appointments. Apart from showing up for the follow-up appointments, participants do not need to adhere to specific tasks.

### Relevant concomitant care permitted or prohibited during the trial {11d}

Not applicable, there is no relevant concomitant care that is permitted or prohibited.

### Provisions for post-trial care {30}

The sponsor has an insurance which is in accordance with the legal requirements in the Netherlands (Article 7 WMO). This insurance provides cover for damage to research participants through injury or death caused by the study. The insurance applies to the damage that becomes apparent during the study or within 4 years after the end of the study.

### Outcomes {12}

**Fig. 1:**
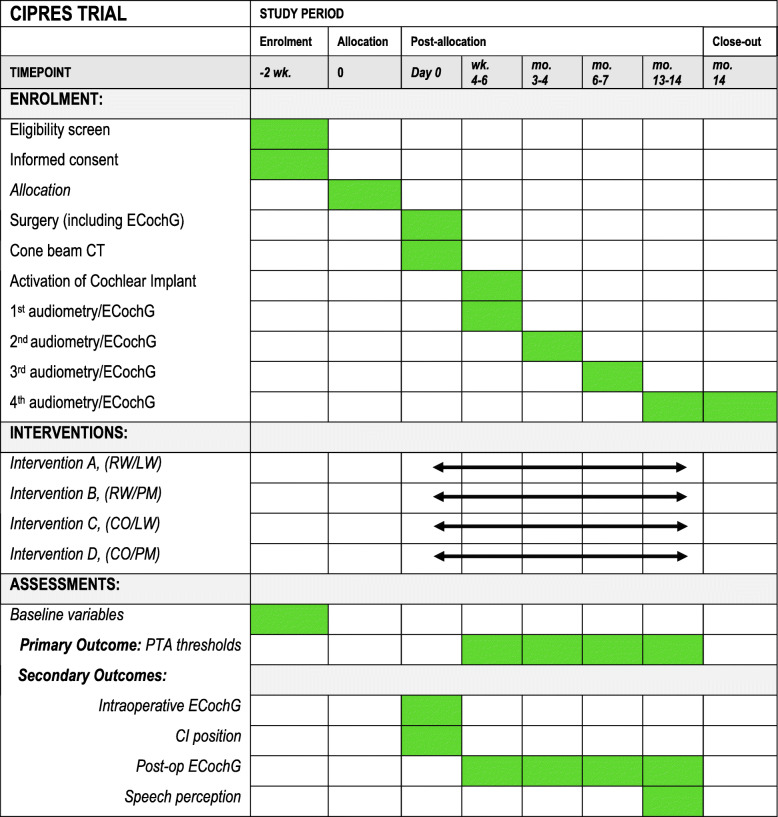
Schedule of enrolment, interventions, and assessments adapted from the Standard Protocol Items: Recommendations for Interventional Trials (SPIRIT)

At intake, baseline data will be collected, including gender, age, duration of deafness, pre- or post-lingually deafened, cause of deafness, and side of implantation. In addition, the most recent pure tone thresholds (250 Hz, 500 Hz, and 1, 2, 4, and 8 kHz) and speech reception thresholds (SRT) for consonant-vowel-consonant (CVC) word lists in quiet for both ears will be collected. See Fig. 1 for the time schedule of all outcomes.

#### Hearing preservation (primary outcome)

Hearing preservation is calculated by comparing pure tone thresholds after and before CI surgery using the following equation [31], Eq. 1:

$$ HP=1-\frac{\left( PTA\  post- PTA\  pre\right)\kern0.75em }{\left( PTA max- PTA\  pre\right)} $$

In this equation, *HP* is hearing preservation; *PTApre* is the average pure tone (unaided) hearing threshold of 125, 250, and 500 Hz measured preoperatively; *PTApost* is the same average pure tone hearing threshold measured postoperatively; and *PTAmax* is the maximum sound intensity generated by a standard audiometer (usually between 90 and 120 dB HL). With full preservation of hearing, *HP* = 100, and with complete loss of hearing *HP* = 0. The postoperative tone audiometry will be measured at 1, 3, 6, and 12 months after activation of the cochlear implant. The primary outcome measure is the average hearing preservation over the four follow-up measurements.

#### Secondary outcomes

##### Scalar positioning of the electrode array

We will use the CB-CT scanner (Newtom VGi EVO, Cefla Italy) to postoperatively assess the scalar location of the electrode array in cochleas of all four groups. The CB-CT has been proven to be the best imaging modality to date, for assessing the scalar location postoperatively, as it has low radiation artifacts (caused by the metal parts of the cochlear implant) and high spatial resolution needed to image the cochlea and its internal parts. Other advantages of this modality are among others that it has relatively low radiation exposure, is less likely to trigger claustrophobic events, and requires shorter scanning durations compared to traditional CT scanners [32–34]. CB-CT imaging postoperatively leads to exposure of low-dose radiation (effective dose 0.18 mSv) and is therefore considered to be of low risk.

We will assess CI translocation by making multiplanar midmodiolar reconstructions of the CB-CT images, which is validated [23–25]. These multiplanar reconstructions will allow us to systematically indicate for every electrode contact of the electrode array the exact scalar position (i.e., scala tympani or scala vestibuli).

##### Electrocochleography

Electrocochleography (ECochG) is a method for recording the electrical potentials of the cochlea. The ECochG is composed of several components: the compound action potential (CAP), auditory nerve neurophonic (ANN), cochlear microphonics (CM), and the summating potential (SP). In essence, the CAP and ANN reflect auditory nerve activity, the CM and SP are generated by the hair cells of the organ of Corti. The CM is an alternating current response following the tone, and the SP is a direct current response. Outcome measures include the total ECochG amplitude. Potentially, the difference in the amplitude of the total ECochG response after and before insertion might contain information about insertion trauma, i.e. damage to the basilar membrane, stria vascularis, or other structures.

Intraoperatively, we will use the most apical contact point of the electrode array to measure these outcomes during insertion. The acoustic pure tone stimuli will be delivered via an earphone (earplug) on the operated ear. This will be coupled to the measurement equipment (active insertion monitoring system, Advanced Bionics) that is provided by the manufacturer. The amplifier in the implant will be used for amplification of the response. Apart from prolonged surgery time (estimate of 10 min), there will be no added risk for the participant. Postoperatively, ECochG measurements will be repeated. We will perform recordings at each of the 16 electrodes for the following frequencies: 125, 250, and 500 Hz and 1, 2, 3, 4 kHz. In addition, acoustic tone thresholds will be indirectly estimated by measures of the total ECochG responses.

##### Speech perception

One year after activation of the CI, a conventional speech perception test in quiet and in noise will be performed with CVC words from the “Nederlandse Vereniging voor Audiologie” (NVA: Dutch Society of Audiology) word-list. Speech reception thresholds will be registered. Also, the clinical spectral ripple test, which uses ripples instead of words, can be used to complement speech perception scores in noise [35].

The extra ECochG measurements and tone/speech tests are not considered to be of any risk for the participants.

### Participant timeline {13}

The schedule of enrolment, interventions, and assessments is depicted in Fig. 1. Participants will be screened and enrolled 2 weeks before the surgery. On the day of surgery, the participant will be allocated to one of the four groups (A–D). During surgery, an intraoperative ECochG measure will be conducted. The CB-CT scan will be performed on the same day after surgery. Activation of the CI is approximately 4–6 weeks after surgery. After activation of the CI, the first audiometry and postoperative ECochG measures will be conducted. These measurements will be repeated at approximately 2, 5, and 12 months after activation of the CI.

### Sample size {14}

Hearing preservation as computed according to Eq. 1 is the primary outcome variable. Sample size calculation was based on a comparison of the means between the four treatment groups using the overall *F* test for an ANOVA. Based on three studies in the literature [36–38], we expect a large range of hearing preservation within each group, from 0 (no preservation, i.e., loss of all hearing) to 100 (full preservation, hearing stable), and occasionally above (improved hearing). In a single group with a mean score of 50, we expect 60% of observations to lie between 25 and 75 points yielding a within-group standard deviation of 30 points assuming a normal distribution. A clinically relevant difference was defined as a difference of 40 points between means in the intervention group with the lowest and highest mean hearing preservation. Assuming means in groups are equally spaced (e.g., 30, 43.3, 56.7, and 70 points), the between-group standard deviation is anticipated to be approximately 17.2 points. The corresponding effect size *f*, found by dividing between-group by within-group standard deviation, equals 0.57. To detect this effect size with 90% power when testing at the 5% significance level, 12 participants need to be included in each of the four treatment groups. The total sample size is set at 14 per intervention group to account for a 10% drop-out rate. Smaller effects can likely be detected because the primary outcome is measured at four different time points for each participant. Assuming a within-subject correlation of 0.5, then the four follow-up measurements per participant will allow detection of effect sizes of *f* = 0.45 and larger. We used G*power (version 3.1.9) to calculate the power.

### Recruitment {15}

No particular strategies were developed to increase the likelihood of participant enrolment. However, in developing the protocol, efforts were made to limit the extra burden for participants participating in this study. For example, follow-up measurements are planned on the same days of rehabilitation appointments. Also, based on previous experience and data, we expect to achieve adequate participant enrolment to reach our targeted sample size.

## Assignment of interventions: allocation

### Sequence generation {16a}

A separate independent department, the Julius Research and Epidemiology Department of the University Medical Center Utrecht, will handle the method of generating the allocation sequence. Randomization will be stratified by age, with two subgroups: 18–50 years and more than 50 years. Every participant will be allocated randomly a number that is generated with a computer, from 4 numbers that are possible (each referring to a unique treatment group). The research tool software of the Julius Center and Epidemiology Department will be used to generate the random sequences. Random sequences will be generated separately for the two age strata. In each age stratum, block randomization is used.

### Concealment mechanism {16b}

The allocation will be done before surgery and after IC approval and screening. Participants will not be informed about the treatment group to which they have been allocated.

### Implementation {16c}

The Julius Center and Epidemiology Department will generate the allocation sequence with their own developed research tool for randomization. A participant can be included by every member of the research team; when in doubt, the inclusion will be judged by the whole research team. Subsequently, based on the allocation, the patient is assigned to one of the groups by a member of the research team.

## Assignment of interventions: blinding

### Who will be blinded {17a}

This study is single-blind, meaning that only participants are blinded for the treatment allocation. Because of the nature of the intervention (type of surgery and intracochlearly placed electrode array), it is impossible for the patient to discover the allocation. The research team is not blinded. The audiology assistants, however, who will perform the audiometry, are blinded. In addition, the offline outcome data will be blindly analyzed.

### Procedure for unblinding if needed {17b}

In rare cases in which the device has to be removed via surgery, unblinding may be permissible. Before the second surgery, the subject will be unblinded by a member of the research team, who will discuss the treatment options with the subject. If needed, the surgeon will also be informed about the exact intervention, as is standard in medical practice.

## Data collection and management

### Plans for assessment and collection of outcomes {18a}

All data will be collected using an electronic data capture (EDC) tool (Castor EDC). The UMC Utrecht healthcare data of the participants, including baseline outcomes, CT images, and results of the audiometry, will be derived from the electronic patient file. The assessors are specialized in otorhinolaryngology, and therefore, they are trained in assessing the audiology, CT images, and electrophysiology data of this study.

### Plans to promote participant retention and complete follow-up {18b}

Once a participant is enrolled and randomized, the study site will make every reasonable effort to follow the subject for the entire study period. Participants can leave the study at any time for any reason if they wish to do so without any consequences. Participants who withdraw from the study or who terminate the recording sessions prematurely, in the absence of any adverse event, will not be followed. Participant retention will be increased by schedule strategies, e.g., by planning the follow-up measures on the same day of clinical rehabilitation. Participants will also be reminded of the study via e-mail between sessions, including information of any published results (if they are interested).

### Data management {19}

All data will be handled confidentially and research data will be coded by using a unique patient identification number. To be able to reproduce the study finding and to help future users to understand and reuse the data all changes made to the raw data and all steps taken in the analysis will be documented. The database files will be kept for 15 years after the study has ended.

### Confidentiality {27}

The key to the code will be safeguarded by the investigators. All data will be stored on the research network disc of the UMC Utrecht in a secured research folder structure. Only the team of investigators will have access to the database files.

### Plans for collection, laboratory evaluation, and storage of biological specimens for genetic or molecular analysis in this trial/future use {33}

Not applicable; no biological specimens will be collected for this study.

## Statistical methods

### Statistical methods for primary and secondary outcomes {20a}

We will use linear mixed models to compare the primary outcome measure (hearing preservation) between the four treatment groups. Linear mixed models will include a random effect for the participant and fixed effects for the intervention group and follow-up visit. In case the overall *F* test for comparing the intervention groups is significant, we will compare means in intervention groups pairwise through posthoc tests using a Bonferroni correction. Fisher’s exact test will be used to compare the proportion of patients with correct electrode location within the scala tympani (correct location after insertion) between the four intervention groups. If the test comparing four groups is significant, then proportions will be compared between each pair of treatment groups separately by means of Fisher’s exact tests and accounting for multiple testing through the use of a Bonferroni correction.

Secondary analyses will include testing of the main effects of surgery type and electrode array type and their two-way interaction. For hearing preservation outcomes, this will be done using linear mixed models. For scalar translocation outcomes, we will use logistic regression. We will also use linear mixed model analysis to identify independent additional predictors for hearing preservation. Among the factors to examine are insertion depth and cochlear volume.

We will use a Pearson correlation test to quantify the strengths of association between ECochG responses and hearing preservation at the various time points (during and after cochlear implantation).

All analyses will be done on an intention-to-treat basis. A two-sided significance level of 5% will be used. The normality assumption for the residuals will be assessed visually using normal-probability plots. In case the normality assumption does not hold, we will use either a transformation of the outcome or an appropriate non-parametric test for comparing the continuous outcomes between treatment groups.

### Interim analyses {21b}

Independent analysis of the Institutional Review Board (IRB) or in Dutch “Medisch Ethische Toetsing Commissie” (METC) classified this study as a low risk, not needing a data safety monitoring committee (DSMC), mainly because all interventions are standard medical care. Therefore, no interim analyses will be conducted during this trial.

### Methods for additional analyses (e.g., subgroup analyses) {20b}

Participants’ age might play an important role in outcomes; however, the randomization procedures are stratified for age which minimizes confounding by age. We will test for an interaction effect of the electrode type and surgical approach using multivariable linear and logistic regression.

### Methods in analysis to handle protocol non-adherence and any statistical methods to handle missing data {20c}

Participants who withdraw from the study or who terminate the recording session prematurely will be considered as lost and will be replaced. Reasons for withdrawal or premature termination will be documented. We expect a withdrawal rate of participants of no more than 10% (since *N* = 48, this is 2 per group). The number of replacements will be limited to two persons per treatment group. Missing at random assumption will be made for the linear mixed model analyses. Depending on the missing values, multiple imputation or simply list-wise deletion will be conducted for the missing values in linear and logistic regression analysis.

### Plans to give access to the full protocol, participant-level data, and statistical code {31c}

Data sharing, including the full protocol, participant datasets, and statistical codes, will be considered upon reasonable request.

## Oversight and monitoring

### Composition of the coordinating center and trial steering committee {5d}

Trial quality will be independently monitored by a local monitor (UMC Utrecht) once a year. The local monitor will check at least 10% of the signed ICs. From the first five participants, the inclusion and exclusion criteria will also be checked. The monocenter study file will be also monitored. This study has no public involvement group.

### Composition of the data monitoring committee, its role, and reporting structure {21a}

Not applicable, a data monitoring committee is not appointed for this study.

### Adverse event reporting and harms {22}

Adverse events (AEs) will be recorded; serious adverse events (SAEs) will be reported to the local IRB and centrally stored in a digital database. Serious adverse events are not expected, but in case they do occur, the research group can decide to terminate prematurely the study.

### Frequency and plans for auditing trial conduct {23}

The investigators will submit a summary of the progress of the trial to the accredited IRB once a year. Information will be provided on the date of inclusion of the first subject, numbers of participants included and numbers of participants that have completed the trial, serious adverse events/serious adverse reactions, protocol violations, other problems, and amendments.

### Plans for communicating important protocol amendments to relevant parties (e.g., trial participants, ethical committees) {25}

Amendments are changes made to the research after a favorable opinion by the accredited IRB has been given. All protocol amendments will be notified to the IRB for approval. Non-substantial amendments will not be notified to the accredited IRB and the competent authority, but will be recorded and filed by the sponsor.

### Dissemination plans {31a}

The trial results will be made accessible to the public in a peer-review journal, preferable in an open access-study journal. In addition, key trial results will be presented in national and international conferences and other relevant meetings. There are no publication restrictions.

## Discussion

Cochlear implantation by way of a cochleostomy or round window approach, using different electrode array types, is the standard medical care for patients with severe to profound bilateral sensorineural hearing loss, as it is a relatively simple and low-risk procedure that greatly benefits patients. Despite the increased interest in hearing preservation, loss of residual hearing remains an important problem in cochlear implantations. This might be caused by a lack of adequate, randomized, and blinded prospective studies, investigating hearing preservation in CI patients. There are studies that investigated hearing preservation in CI patients; however, these studies have a high risk of bias. Therefore, the level of evidence for many aspects of hearing preservation is low. This trial is the first prospective, randomized controlled trial that evaluates the effect of cochlear insertion trauma of several CI treatment options on hearing preservation. Another strength of this study is the evaluation of insertion trauma by three separate assessment tools: audiometry, electrophysiology, and CT imaging. These tools can complement each other, potentially leading even to detection of minimal insertion trauma. In addition, the multiple outcome measures allow us to investigate insertion trauma on the short and long term.

## Trial status

Protocol version 3, 02-01-2020. Date of first recruitment: 31-01-2020. Currently, 3/48 participants are included, date: 12-06-2020. Approximate date of trial completion: 31-01-2023.

## Supplementary Information

**Additional file 1:.** Information letter

## Abbreviations

AE: Adverse event
CB-CT: Cone beam computed tomography
CO: Cochleostomy approach
CI: Cochlear implant
CVC: Consonant-vowel nucleus-consonant, Dutch speech perception test
ECochG: Intracochlear electrocochleography
IC: Informed consent
IRB: Institutional Review Board
LW: Lateral wall electrode array
METC: Medical research ethics committee (MREC); in Dutch: medisch ethische toetsing commissie (METC)
MPT: Mastoidectomy-posterior tympanotomy
PTA: Pure tone audiometry
PM: Perimodiolar electrode array
RW: Round window approach
(S)AE: (Serious) adverse event
SRT: Speech reception threshold
SNHL: Sensorineural hearing loss
WMO: Medical Research Involving Human Participants Act; in Dutch: Wet Medisch-wetenschappelijk Onderzoek met Mensen
