# Supplementary material for: Evaluating cochlear insertion trauma and hearing preservation after cochlear implantation (CIPRES): a study protocol for a randomized single-blind controlled trial
Source: Trials. 2021 Dec 9;22:895. doi: 10.1186/s13063-021-05878-2 (PMC8656003; doi:10.1186/s13063-021-05878-2)
Supplement: Supplementary file 1 — Additional file 1:. Information letter [file 13063_2021_5878_MOESM1_ESM.pdf]

Proefpersoneninformatie voor deelname aan medisch-wetenschappelijk onderzoek

Het verbeteren van de cochleaire implantatie operatie

Gehoorsparend cochleair implanteren

Inleiding

Geachte heer/mevrouw,

Wij vragen u om mee te doen aan een medisch-wetenschappelijk onderzoek.
Meedoen is vrijwillig. Om mee te doen is wel uw schriftelijke toestemming nodig. U ontvangt deze brief omdat u slechthorend bent en u binnenkort hiervoor een cochleair implantaat ontvangt.
Voordat u beslist of u wilt meedoen aan dit onderzoek, krijgt u uitleg over wat het onderzoek inhoudt. Lees deze informatie rustig door en vraag de onderzoeker uitleg als u vragen heeft. U kunt ook de onafhankelijk deskundige, die aan het eind van deze brief genoemd wordt, om aanvullende informatie vragen. U kunt er ook over praten met uw partner, vrienden of familie.
Verdere informatie over meedoen aan zo'n onderzoek staat in de bijgevoegde brochure 'Medisch-wetenschappelijk onderzoek'.

1. Algemene informatie

Dit onderzoek wordt verricht door artsen en medewerkers van de afdeling KNO van het Universitair Medisch Centrum Utrecht. Het onderzoek wordt gesubsidieerd door Advanced Bionics.
De medisch-ethische toetsingscommissie heeft dit onderzoek goedgekeurd. Algemene informatie over de toetsing van onderzoek vindt u in de brochure 'Medisch-wetenschappelijk onderzoek'.

2. Doel van het onderzoek

Er bestaan twee verschillende technieken voor het implanteren van een cochleaire implantaat (CI), en twee soorten implantaten. Bij cochleaire implantatie wordt altijd geopereerd, en gekozen voor één van twee technieken, en voor één van twee typen implantaten. In dit onderzoek vergelijken we het behoud van restgehoor voor deze groepen. De resterende functie van het gehoororgaan dient zoveel mogelijk gespaard te blijven bij het plaatsen van het CI. Het doel van dit onderzoek is

uitzoeken bij welke techniek en bij welk type implantaat dit restgehoor het best gespaard kan worden. We verkrijgen de resultaten aan de hand van metingen tijdens en na de operatie.

3. Achtergrond van het onderzoek

Cochleaire implantatie is een behandeling voor slechthorende patiënten, waarbij de ‘normale’ hoortoestellen geen oplossing bieden. Er bestaan op dit moment twee soorten technieken voor het inbrengen van het implantaat in het slakkenhuis en twee soorten cochleaire implantaten, waarbij het vooralsnog onduidelijk is welke behandeling het beste resultaat biedt. Beide technieken en beide implantaten zijn veilig, ze worden in het UMC Utrecht gebruikt, en worden al meerdere jaren overal in de wereld toegepast.

4. Wat meedoen inhoudt

Operatie

De helft van de proefpersonen krijgt een cochleaire implantaat via een ‘cochleostomie’ techniek en de andere helft via een ‘ronde venster’ techniek. Daarnaast krijgt de ene helft een ‘lateral wall’ implantaat en de andere helft een ‘pre-curved’ implantaat. Loting bepaalt welke combinatie u krijgt.

Bezoeken en metingen

Zie schema in **bijlage B** voor alle onderzoeken.

Anders dan bij de gebruikelijke zorg:

Bij de standaardbehandeling bepaalt de chirurg welke techniek wordt gekozen, dat wordt voor dit onderzoek van te voren bepaald door de loting.

Voor het onderzoek wordt een CT-scan na de operatie verricht van de plek waar de CI is ingebracht. Een CT scan is nodig om te bepalen of het implantaat zich op de correcte plek bevindt in het slakkenhuis. Hierdoor kunnen we de resultaten van de gehoortesten beter beoordelen. Nadeel is dat u, hoewel minimaal, straling ondervindt. Daarnaast wordt ook een extra meting uitgevoerd (elektrocochleografie) tijdens de operatie en 1, 3, 6 en 12 maanden na de operatie. Dit is een draadloze meting met het implantaat, waarbij u tonen te horen krijgt en u niets hoeft te doen.

Bovendien krijgt u vier toonpiepjes testen (vergelijkbaar met de testen vóór de operatie), in plaats van de reguliere twee testen als u niet meedoet met het

onderzoek. En om te bepalen hoe goed uw gehoor is, worden na 12 maanden testen met verschillende geluiden (onder andere toonpiepjes) uitgevoerd.

Standaard medische zorg bij CI

Verder is de reguliere zorg hetzelfde als bij een persoon die een CI krijgt en niet meedoet met het onderzoek. De eerste keer dat u naar het functiecentrum komt, rond 3 tot 4 weken na de operatie, wordt het apparaat voor het eerst aangezet. Vervolgens zal de audioloog het apparaat afstellen op uw specifieke situatie. Het afstellen gebeurt nog driemaal. U komt ook driemaal bij de logopedist en één keer bij de maatschappelijk werker. Deze bezoeken worden gecombineerd met de bezoeken nodig voor deze studie.

5. Wat wordt er van u verwacht

Om het onderzoek goed te laten verlopen, is het belangrijk dat u zich aan de volgende afspraken houdt.

De afspraken zijn dat u:

- afspraken voor bezoeken nakomt.

Het is belangrijk dat u contact opneemt met de onderzoeker:

- als u in een ziekenhuis wordt opgenomen of behandeld.
- als u niet meer wilt meedoen aan het onderzoek.
- als uw contactgegevens wijzigen.

6. Mogelijke ongemakken

Metingen

Over het algemeen wordt de meting met het cochleair implantaat (elektrocochleografie) niet als onaangenaam ervaren. Soms moeten we echter het volume van het aangeboden geluid naar beneden afstellen voor uw gemak.

Stralingsbelasting

Beeldvormende onderzoeken zoals CT-scans maken gebruik van röntgenstraling. Deze onderzoeken zijn nodig om binnen in uw lichaam te kijken. Er bestaat een zeer kleine kans dat de gebruikte straling leidt tot gezondheidsschade. Dit is een acceptabel risico.

7. Mogelijke voor- en nadelen

Het is belangrijk dat u de mogelijke voor- en nadelen goed afweegt voordat u besluit mee te doen. De combinatie van behandeling is niet anders dan de standaardzorg. Door de extra hoortesten en CT scan kunnen we wel een betere inschatting maken van het functioneren met implantaat op de langere termijn.

Nadelen van meedoen aan het onderzoek kunnen zijn :

- mogelijke ongemakken van de metingen in het onderzoek

Deelname aan het onderzoek betekent ook:

- dat u extra tijd kwijt bent (180 minuten over periode van 1 jaar)
- 1 extra afspraak (op maand 6 na operatie)

Al deze zaken zijn hiervoor onder punt 4, 5 en 6 beschreven.

8. Als u niet wilt meedoen of wilt stoppen met het onderzoek

U beslist zelf of u meedoet aan het onderzoek. Deelname is vrijwillig.
Als u niet wilt meedoen, wordt u op de gebruikelijke manier behandeld en krijgt u een cochleaire implantaat.

Als u wel meedoet, kunt u zich altijd bedenken en toch stoppen, ook tijdens het onderzoek. U wordt dan weer op de gebruikelijke manier behandeld voor bij een cochleaire implantaat. U hoeft niet te zeggen waarom u stopt. Wel moet u dit direct melden aan de onderzoeker.
De gegevens die tot dat moment zijn verzameld, worden gebruikt voor het onderzoek.
Als er nieuwe informatie over het onderzoek is die belangrijk voor u is, laat de onderzoeker dit aan u weten. U wordt dan gevraagd of u blijft meedoen.

9. Einde van het onderzoek

Uw deelname aan het onderzoek stopt als

- alle bezoeken volgens het schema in bijlage B voorbij zijn
- u zelf kiest om te stoppen
- de onderzoeker het beter voor u vindt om te stoppen
- Universitair Medisch Centrum Utrecht, de overheid of de beoordelende medisch-ethische toetsingscommissie, besluit om het onderzoek te stoppen.

Het hele onderzoek is afgelopen als alle deelnemers klaar zijn.

Na het verwerken van alle gegevens informeert de onderzoeker u over de belangrijkste uitkomsten van het onderzoek. De onderzoeker kan u ook vertellen in welke groep u zat. Als u dit niet wilt, dan kunt u dit tegen de onderzoeker zeggen. Hij mag het u dan niet vertellen.

10. Gebruik en bewaren van uw gegevens

Voor dit onderzoek worden uw persoonsgegevens gebruikt en bewaard. Het gaat om gegevens zoals uw naam, adres, geboortedatum en om gegevens over uw gezondheid. Het verzamelen, gebruiken en bewaren van uw gegevens is nodig om de vragen die in dit onderzoek worden gesteld te kunnen beantwoorden en de resultaten te kunnen publiceren. Wij vragen voor het gebruik van uw gegevens uw toestemming.

Vertrouwelijkheid van uw gegevens

Om uw privacy te beschermen krijgen uw gegevens een code. Uw naam en andere gegevens die u direct kunnen identificeren worden daarbij weggelaten. Alleen met de sleutel van de code zijn gegevens tot u te herleiden. De sleutel van de code blijft veilig opgeborgen in de lokale onderzoeksinstelling. Ook in rapporten en publicaties over het onderzoek zijn de gegevens niet tot u te herleiden.

Toegang tot uw gegevens voor controle

Sommige personen kunnen op de onderzoekslocatie toegang krijgen tot al uw gegevens. Ook tot de gegevens zonder code. Dit is nodig om te kunnen controleren of het onderzoek goed en betrouwbaar is uitgevoerd. Personen die ter controle inzage krijgen in uw gegevens zijn: de commissie die de veiligheid van het onderzoek in de gaten houdt, een controleur die voor de onderzoeker van het onderzoek werkt. Zij houden uw gegevens geheim. Wij vragen u voor deze inzage toestemming te geven.

Bewaartermijn gegevens

Uw gegevens moeten 15 jaar worden bewaard na afloop van het onderzoek.
Het wordt bewaard om daarop in de loop van dit onderzoek nog nieuwe bepalingen te kunnen doen die te maken hebben met dit onderzoek.

Bewaren en gebruik van gegevens voor ander onderzoek

Uw gegevens kunnen na afloop van dit onderzoek ook nog van belang zijn voor ander wetenschappelijk onderzoek op het gebied van de ontwikkeling van cochleaire

implantaten. Daarvoor zullen uw gegevens 15 jaar worden bewaard na afloop van het onderzoek. U kunt op het toestemmingsformulier aangeven of u hier wel of niet mee instemt. Indien u hier niet mee instemt, kunt u gewoon deelnemen aan het huidige onderzoek.

Informatie over onverwachte bevindingen

Wij verwachten geen onverwachte bevindingen. Indien dat wel het geval is en het is belangrijk voor uw gezondheid, dan zult u op de hoogte worden gesteld door de specialist. U kunt dan met uw huisarts of specialist bespreken wat er gedaan moet worden. Ook hiervoor geeft u toestemming.

Intrekken toestemming

U kunt uw toestemming voor gebruik van uw persoonsgegevens altijd weer intrekken. Dit geldt voor dit onderzoek en ook voor het bewaren en het gebruik voor het toekomstige onderzoek. De onderzoeksgegevens die zijn verzameld tot het moment dat u uw toestemming intrekt worden nog wel gebruikt in het onderzoek.

Meer informatie over uw rechten bij verwerking van gegevens

Voor algemene informatie over uw rechten bij verwerking van uw persoonsgegevens kunt u de website van de Autoriteit Persoonsgegevens raadplegen. De website van het UMC Utrecht: www.umcutrecht.nl

Bij vragen over uw rechten kunt u contact opnemen met de verantwoordelijke voor de verwerking van uw persoonsgegevens. Voor dit onderzoek is dat:

Universitair Medisch Centrum Utrecht, zie **bijlage A** voor contactgegevens.

Bij vragen of klachten over de verwerking van uw persoonsgegevens raden we u aan eerst contact op te nemen met de onderzoek locatie. U kunt ook contact opnemen met de Functionaris voor de Gegevensbescherming van de instelling of de Autoriteit Persoonsgegevens.

11. Verzekering voor proefpersonen

Voor iedereen die meedoet aan dit onderzoek is een verzekering afgesloten. De verzekering dekt schade als gevolg van het onderzoek. Dit geldt voor schade die naar boven komt tijdens het onderzoek, of binnen vier jaar na het einde van het onderzoek.

12. Geen vergoeding voor meedoen

De extra testen en behandeling voor het onderzoek kosten u niets. U wordt niet betaald voor het meedoen aan dit onderzoek. Wel krijgt u een vergoeding voor uw (extra) reiskosten.

13. Heeft u vragen?

Bij vragen kunt u contact opnemen met het onderzoeksteam. Voor onafhankelijk advies over meedoen aan dit onderzoek kunt u terecht bij de onafhankelijke arts. Hij weet veel over het onderzoek, maar heeft niets te maken met dit onderzoek.

Indien u klachten heeft over het onderzoek, kunt u dit bespreken met de onderzoeker of uw behandelend arts. Wilt u dit liever niet, dan kunt u zich wenden tot de klachtencommissie van uw ziekenhuis. Alle gegevens vindt u in **bijlage A**: Contactgegevens.

14. Ondertekening toestemmingsformulier

Wanneer u voldoende bedenktijd heeft gehad, wordt u gevraagd te beslissen over deelname aan dit onderzoek. Indien u toestemming geeft, zullen wij u vragen deze op de bijbehorende toestemmingsverklaring schriftelijk te bevestigen. Door uw schriftelijke toestemming geeft u aan dat u de informatie heeft begrepen en instemt met deelname aan het onderzoek.

Zowel uzelf als de onderzoeker ontvangen een getekende versie van deze toestemmingsverklaring.

Dank voor uw aandacht

16. Bijlagen bij deze informatie

A. Contactgegevens
B. Schema onderzoek
C. Informatie over verzekering
D. Toestemmingsformulier proefpersoon

Bijlage [A]: contactgegevens voor Universitair Medisch Centrum Utrecht

Hoofdonderzoeker: Dr. H.G.X.M Thomeer, Department of Otorhinolaryngology and Head & Neck Surgery, University Medical Center Utrecht, Heidelberglaan 100, 3584 CX Utrecht, Netherlands. Email: h.g.x.m.thomeer@umcutrecht.nl

Onafhankelijk arts: Dr. A.J.N Bittermann, Department of Otorhinolaryngology and Head & Neck Surgery, University Medical Center Utrecht, Heidelberglaan 100, 3584 CX Utrecht, Netherlands. Email: A.J.N.Bittermann@umcutrecht.nl

Uitvoerende onderzoeker: Drs. S. Jwair, Department of Otorhinolaryngology and Head & Neck Surgery, University Medical Center Utrecht, Heidelberglaan 100, 3584 CX Utrecht, Netherlands. Email: s.jwair@umcutrecht.nl

Klachten: Klachtenbemiddeling UMC Utrecht, Huispost D01.343, Antwoordnummer 8419, Postbus 85500, 3508 GA UTRECHT, Telefoon 088 75 562 08

Functionaris voor de Gegevensbescherming van de instelling:
privacy@umcutrecht.nl, UMC Utrecht t.a.v. Functionaris gegevensbescherming, Huispostnummer Fac. 10.12, Postbus 85500, 3508GA Utrecht,

Alarmnummer/24-uur bereikbaar: afdeling KNO via tlf. UMC Utrecht: 088 755 5555

Bijlage [B] Schema onderzoek

De extra metingen voor dit onderzoek worden gecombineerd met de regulieren afspraken.

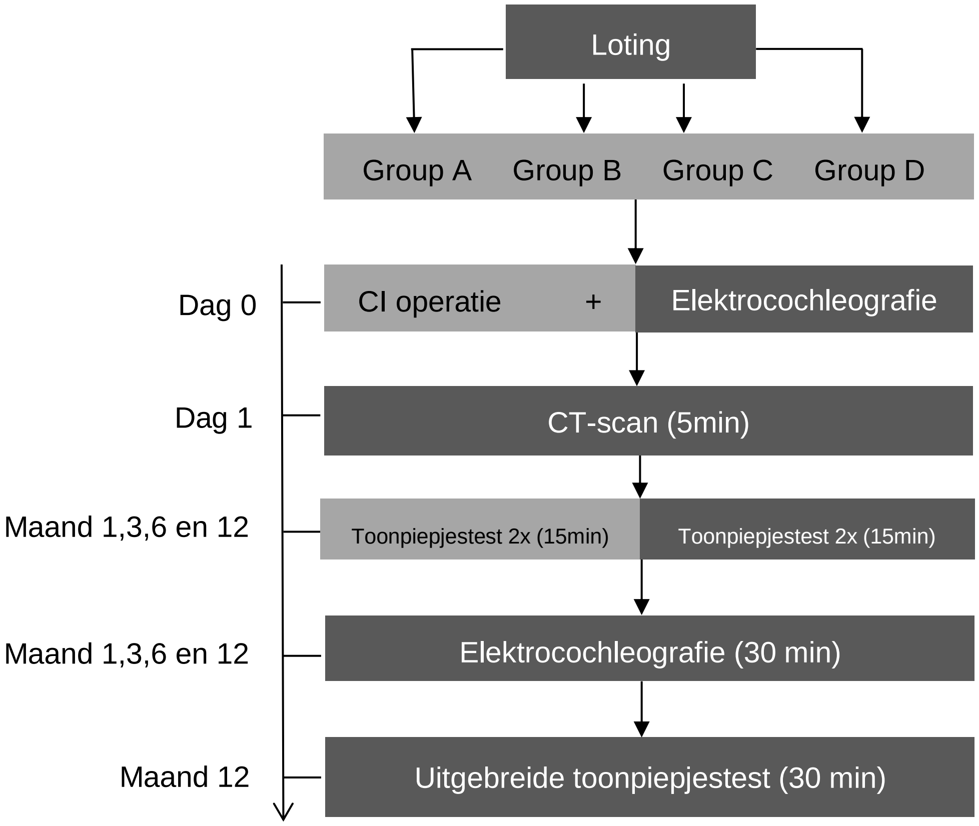

Licht grijs: Standaard medische zorg bij CI
Donker grijs: Extra voor studie

Bijlage [C]: informatie over de verzekering

Voor iedereen die meedoet aan dit onderzoek, heeft universitair medische centrum Utrecht een verzekering afgesloten. De verzekering dekt schade door deelname aan het onderzoek. Dit geldt voor schade tijdens het onderzoek of binnen vier jaar na het einde van uw deelname aan het onderzoek. Schade moet u binnen die vier jaar aan de verzekeraar hebben gemeld.

De verzekering dekt niet alle schade. Onderaan deze tekst staat in het kort welke schade niet wordt gedekt.
Deze bepalingen staan in het 'Besluit verplichte verzekering bij medisch-wetenschappelijk onderzoek met mensen 2015'. Dit besluit staat in de Wettenbank van de overheid (https://wetten.overheid.nl).

Bij schade kunt u direct contact leggen met de verzekeraar [of schaderegelaar].

De verzekeraar van het onderzoek is:<br>Naam:<br>Adres:<br>Telefoonnummer:<br>E-mail: …<br>(Polisnummer: …)<br>(Contactpersoon: …)

De verzekering biedt een dekking van maximaal 650.000 per proefpersoon en maximaal 5.00.000 voor het hele onderzoek en maximaal 7.500.000 per jaar voor alle onderzoeken van dezelfde opdrachtgever.

De verzekering dekt de volgende schade **niet**:

- schade door een risico waarover u in de schriftelijke informatie bent ingelicht. Dit geldt niet als het risico zich ernstiger voordoet dan was voorzien of als het risico heel onwaarschijnlijk was;
- schade aan uw gezondheid die ook zou zijn ontstaan als u niet aan het onderzoek had meegedaan;
- schade door het niet (volledig) opvolgen van aanwijzingen of instructies;
- schade aan uw nakomelingen, als gevolg van een negatief effect van het onderzoek op u of uw nakomelingen;

- schade door een bestaande behandelmethode bij onderzoek naar bestaande behandelmethoden.

Bijlage [D]: toestemmingsformulier proefpersoon

Het verbeteren van de cochleaire implantatie operatie

- Ik heb de informatiebrief gelezen. Ook kon ik vragen stellen. Mijn vragen zijn voldoende beantwoord. Ik had genoeg tijd om te beslissen of ik meedoe.
- Ik weet dat meedoen vrijwillig is. Ook weet ik dat ik op ieder moment kan beslissen om toch niet mee te doen of te stoppen met het onderzoek. Daarvoor hoef ik geen reden te geven.
- Ik geef toestemming voor het informeren van mijn huisarts dat ik meedoe aan dit onderzoek
- Ik geef toestemming voor het verzamelen en gebruiken van mijn gegevens voor de beantwoording van de onderzoeksvraag in dit onderzoek.
- Ik weet dat voor de controle van het onderzoek sommige mensen toegang tot al mijn gegevens kunnen krijgen. Die mensen staan vermeld in deze informatiebrief. Ik geef toestemming voor die inzage door deze personen.
- Ik geef □ **wel**

  □ **geen**

  toestemming om mijn persoonsgegevens langer te bewaren en te gebruiken voor toekomstig onderzoek op het gebied van behandeling van gehoorverlies.
- Ik geef □ **wel**

  □ **geen**

  toestemming om mij na dit onderzoek opnieuw te benaderen voor een vervolgonderzoek.
- Ik wil meedoen aan dit onderzoek.

Naam proefpersoon:

Handtekening: Datum : __ / __ / __

-

Ik verklaar dat ik deze proefpersoon volledig heb geïnformeerd over het genoemde onderzoek.

Als er tijdens het onderzoek informatie bekend wordt die de toestemming van de proefpersoon zou kunnen beïnvloeden, dan breng ik hem/haar daarvan tijdig op de hoogte.

De behandelend specialist wordt bij toestemming geïnformeerd.

Naam onderzoeker (of diens vertegenwoordiger):

Handtekening: Datum: __ / __ / __

-

De proefpersoon krijgt een volledige informatiebrief mee, samen met een getekende versie van het toestemmingsformulier.
